# Isolation and molecular identification of the etiological agents of streptococcosis in Nile tilapia (*Oreochromis niloticus*) cultured in net cages in Lake Sentani, Papua, Indonesia

**DOI:** 10.1186/2193-1801-3-627

**Published:** 2014-10-24

**Authors:** Hilal Anshary, Rio A Kurniawan, Sriwulan Sriwulan, Ramli Ramli, Dolores V Baxa

**Affiliations:** Laboratory of Fish Parasites and Diseases, Department of Fisheries, Faculty of Marine Science and Fisheries, Hasanuddin University, Makassar, Indonesia; Fish Quarantine and Inspection Agency Regional Jayapura, Ministry of Marine Affairs and Fisheries, Jakarta, Republic of Indonesia; School of Veterinary Medicine, Department of Anatomy, Physiology, and Cell Biology, University of California, Davis, CA 95616 USA

**Keywords:** Streptococcosis, Nile tilapia, Cage, Lake Sentani, *Streptococcus*, *Lactococcus garvieae*

## Abstract

Infections with *Streptococcus* spp. were observed in Nile tilapia cultured in net cages in Lake Sentani, Papua, Indonesia. Clinical signs included exophthalmia, erratic swimming, ascites in abdominal cavity, and external hemorrhages. Four types of bacterial colonies (SK, K10, P20, and M12) were isolated from the brain, kidney, and eyes. Based on phenotypic and genetic (16S rDNA sequencing) characteristics, the isolates were identified as *Streptococcus iniae* (SK), *Streptococcus agalactiae* (K10 and P20) and *Lactococcus garvieae* (M12). The latter species has not been previously isolated or reported from fish streptococcosis in Indonesia. Intraperitoneal injection of healthy tilapia with the bacterial species caused significant morbidity (70%) within 3 days and 100% mortality at 6 days post injection. Experimental infections and reisolation of the bacteria from morbid and dead fish suggest they are the causative agents of streptococcosis, which rendered high mortality among cage cultured Nile tilapia in Lake Sentani. Our results suggest the need for developing diagnostic tools for accurate identification of the agents of streptococcosis. As tilapia aquaculture continues to expand as a means of food production and livelihood in Indonesia, it becomes crucial to ensure that fish resources are monitored and protected from the adverse effects of infectious diseases.

## Background

Nile tilapia (*Oreochromis niloticus*) has been the focus of major aquaculture efforts worldwide because the fish is easy to cultivate, adapts to a wide range of environmental conditions, grows fast, tolerant to high stocking density, and is relatively resistant to stress and diseases (El-Sayed 2006). Nile tilapia is extensively cultivated in Indonesia particularly in Java, Sumatra, Kalimantan and Sulawesi Islands using various types of culture systems such as pond and net cage cultures in lakes or in dams (Working Group of Marine and Fisheries Data Arrangement 2013). Aquaculture of Nile tilapia in Indonesia has increased considerably from 291,030 MT in 2008 to 695,063 MT in 2012 (Working Group of Marine and Fisheries Data Arrangement 2013), providing an important source of livelihood among communities along the shores of Lake Sentani, Papua, east Indonesia. Although commercial tilapia culture has been a profitable source of income for local fish farmers, infectious diseases have been emerging.

Streptococcosis, a bacterial disease, is one of the most significant diseases of tilapia worldwide. The causative agents of the disease have been attributed to multiple bacterial agents but most commonly *Streptococcus* and/or *Lactococcus* (Kang et al. 2004; Plumb and Hanson 2011). Streptococcosis is a common disease affecting various species of fish worldwide (Roberts 2012) including the Asian seabass (Suanyuk et al. 2010), barramundi (Bromage et al. 1999), grouper (Bowater et al. 2012), Japanese flounder (Nguyen et al. 2002), rabbit fish (Yuasa et al. 1999), rainbow trout (Eldar and Ghittino 1999), red drum (Eldar et al. 1999; Shen et al. 2005), red tilapia (Hernandez et al. 2009; Musa et al. 2009), seabream (Evans et al. 2002), silver pomfret (Duremdez et al. 2004), and wild mullet (Evans et al. 2002). An annual global loss of about 250 million USD has been attributed to streptococcosis (Amal and Zamri-Saad 2011). The clinical signs of streptococcosis or lactococcosis include unilateral or bilateral exophthalmia, erratic swimming, pale gills, opaque cornea, external haemorrhages, enlarged spleen, ascites in abdominal cavities, and discolored liver (Suanyuk et al. 2005; Salati 2011).

Several species of *Streptococcus* and *Lactococcus* have been identified as the cause of streptococcosis/lactococcosis in fish including *Streptococcus iniae*, *S. agalactiae* (synonym *S. difficile*), *S. dysgalactiae*, *S. parauberis*, and *Lactococcus garvieae* (Eldar et al. 1994; Kang et al. 2004; Nomoto et al. 2004; Vendrell et al. 2006; Agnew and Barnes 2007; Soltani et al. 2008; Plumb and Hanson 2011; Abdelsalam et al. 2013). *S. iniae* and *S. agalactiae* have been frequently reported as the causative agents of streptococcosis in tilapia worldwide (Perera et al. 1994; Eldar et al. 1995; Bowser et al. 1998; Salvador et al. 2005; Suanyuk et al. 2005, 2010; Yuasa et al. 2008; Hernandez et al. 2009; Musa et al. 2009; Abuseliana et al. 2010; Lusiastuti et al. 2012; Najiah et al. 2012; Figueiredo et al. 2012). Except for one report from Brazil (Evans et al. 2009), *L. garvieae* has been rarely isolated in tilapia with streptococcosis. *Enterococcus seriolicida*, the synonym of *L. garvieae* (Eldar et al. 1996), is the most common causative agent of fish streptococcosis in Japan (Kusuda et al. 1991).

In Indonesia, *S. agalactiae* and *S. iniae* have been implicated in streptococcosis infections in commercial culture of tilapia (Yuasa et al. 2008; Lusiastuti et al. 2012). During a monitoring survey of the health status of tilapia in Lake Sentani from 2010 to 2013, severe morbidity and mortality were observed. Clinical symptoms included long white fecal string, erratic swimming, pale gills, opaque cornea, and exophthalmus. The isolated bacteria were β-haemolytic, Gram positive, non-motile cocci, and presumptively identified as *Streptococcus* sp. Biochemical testing failed to identify the bacteria to the species level. Despite the significant losses of tilapia due to streptococcal infection in Indonesia, few studies are available on the etiology of streptococcosis/lactococcosis in naturally infected fish. Furthermore, a more definitive identification of the causative agents using molecular techniques are currently lacking in Indonesia. For these reasons, the main goal of our study is to identify the causative agent(s) of streptococcosis among cage cultured tilapia in Lake Sentani, Papua, Indonesia using 16S rRNA gene sequencing and phylogenetic analysis. As a secondary objective, healthy tilapia were intraperitoneally inoculated with the bacterial isolates to confirm the etiology of the disease.

## Results

### Bacterial isolation and biochemical characterization

Moribund tilapia (n = 25) were observed at 1–4 months following stocking in cages. Three types of Gram positive bacteria were isolated from the kidney (SK), brain (K10 and P20), and eyes (M12). Type strains were evaluated for biochemical characteristics and compared to *S. iniae, S. agalactiae*, and *L. garvieae* (Table 1). All isolates were cocci in chains, non-motile, catalase negative, oxidase negative, fermentative, and did not grow on MacConkey agar. The SK isolate was β-haemolytic while the rest were α-haemolytic. Differences in biochemical tests among the three bacterial species were observed in esculin, salicin, trehalose, and Voges Proskauer tests. *S. iniae* and *L. garvieae* fermented esculin, lactose, salicin and trehalose, whereas *S. agalactiae* did not ferment the carbohydrates. *L. garvieae* was positive for lactose fermentation. *S. iniae*, in contrast to *S. agalactiae* and *L. garvieae*, showed a negative VP reaction. Except for mannitol utilization, K10 and P20 revealed similar biochemical characteristics with *S. agalactiae* described by Austin and Austin ( 2012). The SK isolate is similar to *S. iniae* in all biochemical characteristics. The M12 isolate was similar to *L. garvieae* except it utilized lactose but not sorbitol (Table 1).

**Table 1:** **Biochemical characteristics of bacterial isolates from Nile tilapia compared with** ***Streptococcus iniae, S. agalactiae*** **and** ***Lactococcus garvieae***

Characteristics	***S. iniae***(Austin and Austin 2012)	***S. agalactiae***(Austin and Austin 2012)	***L. garvieae***(Austin and Austin 2012)	Present Isolates			
				SK	P20	K10	M12
Gram stain	+	+	+	+	+	+	+
Shape	Coccus	Coccus	Coccus	Coccus	Coccus	Coccus	Coccus
Motility	–	–	–	–	–	–	–
Aerobic growth	+	+	+	+	+	+	+
Catalase	–	–	–	–	–	–	–
Oxidase	–	–	–	–	–	–	–
O/F	F	F	F	F	F	F	F
Growth at 10°C	–	–	+	+	+	+	+
Growth at 45°C	–	–	+	–	–	–	–
Growth at 6.5% NaCl	–	–	+	–	–	–	–
Blood hemolysis	α/β	-/d	α	β	α	α	α
Esculin	+	–	+	+	–	–	+
Lactose	–	–	–	–	–	–	+
Mannitol	+	–	+	+	+	+	+
Raffinose	–	–	–	–	–	–	–
Salicin	+	–	+	+	–	–	+
Sorbitol	–	–	+	–	–	–	–
Trehalose	+	–	+	+	–	–	+
VP test	–	+	+	–	+	+	+
Glucose	+	+	+	+	+	+	+
MacConkey Agar	–	–	–	–	–	–	–

### Pathogenicity of isolates

**Figure 1:**
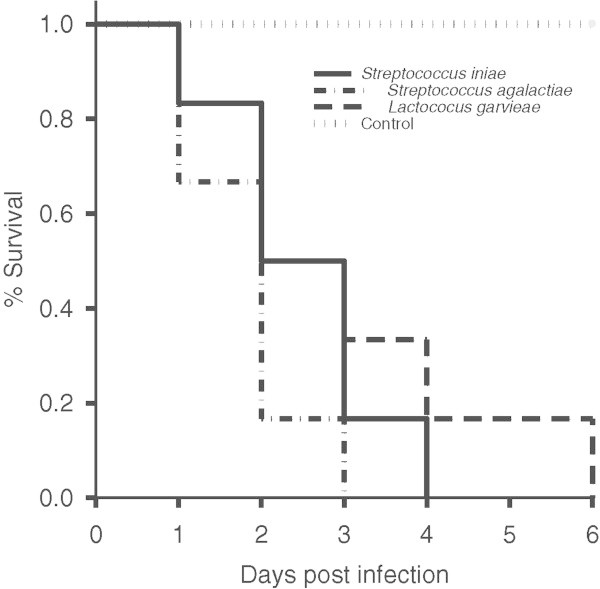
**Kaplan-Meier survival curve of Nile tilapia after challenge with the isolates:** ***Streptococcus iniae*** **(SK),** ***S*** **.** ***agalactiae*** **(K10, P20) and** ***Lactococcus garvieae*** **(M12).** Each fish was intraperitoneally injected with 0.1 ml of each bacterial suspension (1.0 × 10^7^ CFU/mL mean density).

The four isolates that were phenotypically similar to *S. iniae* (SK), *S. agalactiae* (K10 and P20), and *L. garvieae* (M12) showed high pathogenicity to tilapia. Kaplan-Meier survival analysis showed that the mean survival time of tilapia injected with *S. iniae*, *S. agalactiae* and *L. garvieae* (0.1 ml of 1.0 × 10^7^ CFU/mL) was 2.5, 1.83, and 3 days, respectively (Figure 1). All of the fish injected with each suspension of the bacterial isolate showed clinical signs and died within 6 days after injection. Morbidity or mortality was not observed in the control group while the bacteria were recovered from morbid and dead fish in the exposed injected groups.

### Identification of the isolates by molecular methods

The DNA of each isolate was analyzed by PCR using the 16S rRNA universal primers that amplified fragments of approximately 1,500 bp in size. PCR reactions using these primers amplified a 1,482 bp from the SK isolate. Sequence alignments with known sequences in the GenBank database showed that the SK isolate had high similarity (99.8 − 99.9%) to *S. iniae* from red drum in China, Nile tilapia in Thailand, *S. iniae* strain ATCC 29178 from Israel, rainbow trout in Iran, and *S. iniae* from Nile tilapia in Thailand (99.1%). The 1,490 bp fragment from M12 showed a 100% similarity with GenBank sequences of *L. garvieae* from yellowtail in Japan and from rainbow trout in Iran, 99.9% similarity with grey mullet in Taiwan, 99.7% with rainbow trout in Iran, and 99.4% with giant freshwater prawn in Taiwan. The K10 (1,487 bp) and P20 (1,491 bp) isolates showed a 99.8 − 99.9% sequence similarity to *S. agalactiae* from tilapia in China.

**Figure 2:**
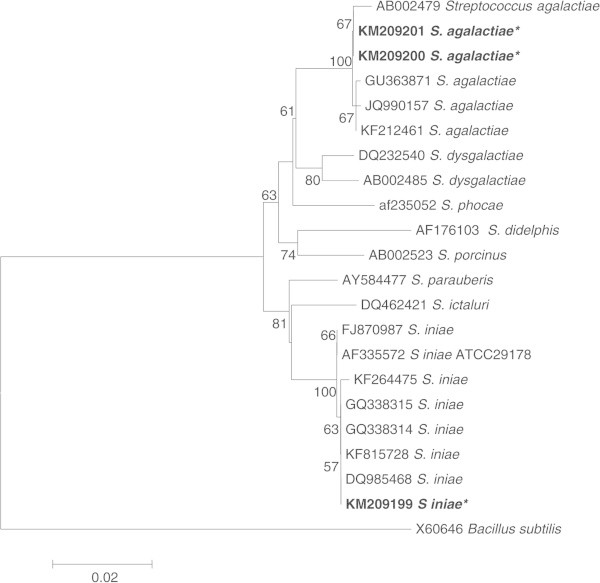
**Phylogenetic tree of** ***Streptococcus iniae*** **(SK − KM209199) and** ***S. agalactiae*** (K10 − KM209200; P20− KM209201) from the present study (*) and other ***Streptococcus*** **spp. based on 16S rRNA gene sequences.** Neighbor-joining tree was constructed using MEGA 6 maximum composite likelihood method and 1000 bootstrap number with complete deletion. Percentages ≥50% are shown at the internal nodes. *Bacillus subtilis* was used as an outgroup.

**Figure 3:**
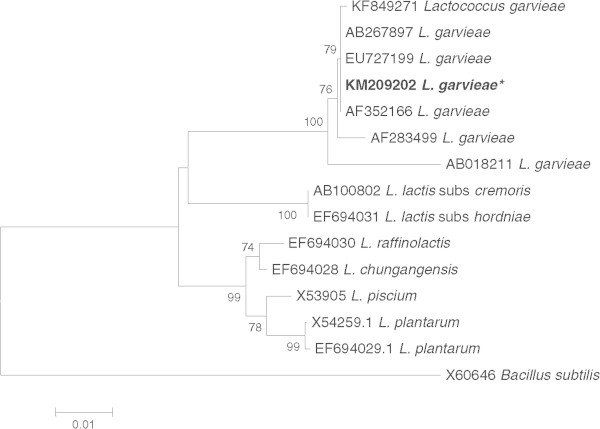
**Phylogenetic tree of** ***Lactococcus garvieae*** (M12− KM209202) from the present study (*) and other ***Lactococcus*** **spp. based on 16S rRNA gene sequences.** Neighbor-joining tree was constructed using MEGA 6 maximum composite likelihood method and 1000 bootstrap number with complete deletion. Percentages ≥50% are shown at the internal nodes. *Bacillus subtilis* was used as an outgroup.

A phylogenetic tree was constructed using the Neighbor-joining method which showed that *S. agalactiae* (K10 and P20) and *S. iniae* (SK) in the present study was placed in the same clade with respective species in GenBank (Figure 2). The same result was observed in the phylogenetic analysis of *L. garvieae* (M12) in that it was placed in the same clade as other *L. garvieae* reported from fish and shrimp (Figure 3).

## Discussion

Streptococcosis is the main disease affecting Nile tilapia and many other fish species globally. Although the disease is widely distributed in Indonesia, particularly in West Java and South Sumatra Provinces, the nature and extent of the disease incidence in the region remain unclear. Previous studies showed that the causative agents of streptococcosis in Indonesia were identified as *S. iniae* using oligonucleotide probe array targeting a specific region in the 16S rRNA (Yuasa et al. 2008) and *S. agalactiae* by multilocus sequence typing (Lusiastuti et al. 2012). The phenotypic characteristics of the present isolates SK, K10, P20, and M12 showed variable carbohydrate utilization when compared to *S. iniae*, *S. agalactiae*, and *L. garvieae* (Austin and Austin 2012). Other studies have shown that *S. iniae*, *S. agalactiae*, and *L. garvieae* isolated from different hosts have considerable diversity in biochemical characteristics including acid production from carbohydrates (Buller 2004; Cagirgan 2004; Garcia et al. 2008; Sharifiyazdi et al. 2010). Most *S. agalactiae* isolated from fish exhibited a negative reaction in mannitol test (Buller 2004; Garcia et al. 2008), but some strains from cattle showed a positive reaction (Garcia et al. 2008). Difference in lactose fermentation has been observed among *L. garvieae* strains. For example, *L. garvieae* from rainbow trout in Iran and *L. garvieae* ATCC 43921 as well as isolates from rainbow trout in Turkey were positive in lactose fermentation (Cagirgan 2004; Sharifiyazdi et al. 2010). As these studies demonstrate, differences in biochemical properties may provide important information on the physiological profile of the organism. However, phenotypic characteristics alone cannot provide a definitive identification to the species level. Molecular methods have the ability to identify pathogenic species and to discriminate to species and strains level (Cunningham 2002) and were therefore used in the current study.

Based on phenotypic and gene sequence analyses, our study identified three species of bacteria (*S. iniae*, *S. agalactiae*, and *L. garvieae*) as the causative agents of streptococcosis/lactococcosis in the Nile tilapia reared in floating net cages in Lake Sentani, Papua. Sequencing the 16S rRNA region and phylogenetic analysis showed high similarities (99.9 – 100%) in nucleotide sequences between the current isolates as compared with specific sequences in GenBank. Based on these results, the isolates from the diseased tilapia in our study were confirmed as *S. iniae* (SK), *S. agalactiae* (K10 and P20) and *L. garvieae* (M12). There has been no previous report on the association and identification of *L. garvieae* in streptococcosis outbreaks in this region. To the best of our knowledge, our studies provide the first report on the isolation of *L. garvieae* among Nile tilapia in Indonesia suffering from streptococcosis. Globally, *S. iniae* and *S. agalactiae* are the most common agents of streptococcosis in tilapia with one report on *L. garvieae* from Brazil (Evans et al. 2009). *Enterococcus seriolicida*, the synonym of *L. garvieae*, is the agent of streptococcosis in yellowtail and eel culture in Japan (Kusuda et al. 1991). Although we have isolated three bacterial species (*S. iniae*, *S. agalactiae* and *L. garvieae*) from cultured Nile tilapia, it is unknown if these pathogens co-existed in an individual fish. Streptococcosis in farm-raised Nile tilapia in Brazil showed *S. agalactiae* and *S. difficile* (Salvador et al. 2005) although the latter was eventually identified to be the same species as *S. agalactiae* based on whole cell protein profiles and high similarity in gene sequences (Vandamme et al. 1997; Kawamura et al. 2005). Infection with *S. parauberis* and *L. garvieae* have been reported from diseased flounder (Baeck et al. 2006) including mixed infection of *S. iniae*, *S. dysgalactiae* and *L. garvieae* in yellowtail (Hussein and Hatai 2006).

Koch’s postulates confirmed *S. iniae*, *S. agalactiae*, and *L. garvieae* as the causative agents of streptococcosis among caged tilapia in Lake Sentani. Morbidity and mortality is predicted to be about 50 – 80% in cage cultured tilapia in the region. Small or larger fish (1 to 4 months post stocking) are apparently susceptible to the disease. Significant mortalities suggest that the seeded tilapia that was raised in hatcheries and then planted into the cages may have been the source of infection even if the fish succumbed to infection later. Although transmission of *Streptococcus* from wild to cultured fish is common (Zlotkin et al. 1998; Evans et al. 2002), the reservoir of infection with *Streptococcus* remains to be determined in Lake Sentani. Juvenile fish seeded into cages normally show signs of streptococcosis after 1 to 2 months of culture. It is unknown if the fish acquired the infection in the hatchery prior to seeding in the cages or were infected during cage culture where the disease progressed due to physicochemical stressors present in the lake. For example, mean water temperature at the time of sampling was 28.5°C in the morning and 30°C at noon. Previous studies suggest that high water temperature enhanced the severity of streptococcosis in cage cultured tilapia (Al-Marzouk et al. 2005; Baeck et al. 2006; Siti-Zahrah et al. 2008; Najiah et al. 2012). Dissolved oxygen, ammonia, turbidity, and pH also affected the progress of infections with *S. agalactiae* in cage culture of hybrid tilapia in lakes (Amal et al. 2013). Disease transmission among all life stages of fish are enhanced by environmental and internal factors (Schreck et al. 1993) and other risk factors such as water quality, pollutants, and fish management activities (Riley et al. 2008). Factors affecting the virulence of the bacteria isolated in the current study are unknown. In *S. agalactiae*, proteins and enzymes linked to cell surface metabolism were found while capsule genes, haemolysin and adhesion genes, were associated as virulence factors in *L. garvieae* (Morita et al. 2011; Miyauchi et al. 2012). Geographic strains also vary in pathogenicity as those observed in *S. agalactiae* (Evans et al. 2014).

Streptococcosis may be transmitted through various routes of infection. Infected fish release bacteria through the feces, survive in the water, and infect other healthy fish (Nguyen et al. 2002). *S. iniae* may be transmitted via oral and olfactory routes in the water column and enhance horizontal transmission of the bacteria (Shoemaker et al. 2000). In Lake Sentani, dead and moribund fish are immediately removed from healthy population. However, dead fish carcasses are recycled as food sources to cultivated species adjacent to the tilapia cage cultures. This practice provides a reservoir of infection to tilapia cultured in floating net cages and source of streptococcosis outbreaks across tilapia populations in the area. Feeding with contaminated diets is a source of streptococcosis outbreaks in flounder in Korea (Kim et al. 2007). Transmission of bacteria from wild fish to cultured fish is also possible (Zlotkin et al. 1998; Evans et al. 2002). *S. agalactiae* isolated from diseased ya-fish (*Schizothorax prenanti*) could be experimentally transmitted to crucian carp (*Carassius carassius*) and Nile tilapia (Geng et al. 2012) suggesting potential horizontal transmission (e.g. fish to fish) among cultured species in the area. Since the bacteria have low host specificity, it is possible that the fish inhabiting the lake are carriers of the pathogen and subsequently act as reservoir of infection of tilapia cultivated in the lake. Together, these potential sources of infection may explain the persistence of streptococcosis among Nile tilapia cultured in Lake Sentani. Further study on the epidemiology of this disease in Lake Sentani may provide information on how to reduce the risks of streptococcosis outbreak in the region.

## Conclusions

Based on phenotypic and genetic characteristics, three species were identified as the causative agents of streptococcosis in tilapia: *S. iniae* (SK), *S. agalactiae* (K10 and P20), and *L. garvieae* (M12). These species showed high pathogenicity in tilapia and were confirmed as the agents of streptococcosis by fulfilling Koch’s postulates. All three species are the most likely cause of significant mortality in caged tilapia in Lake Sentani, Papua. The persistence of these pathogens in cultured tilapia and wild fish will be a bottleneck for intensive culture of tilapia in Lake Sentani. Developing standard and quantitative PCR assays using the specific gene sequences that we obtained in the current study will provide rapid and specific diagnostic techniques for early detection of the pathogen and for estimating the prevalence and distribution of fish infected with streptococcosis. The potential application of a vaccine is another promising area of investigation in the future to combat the spread of the disease. As tilapia aquaculture continues to expand as a means of food production in Indonesia, it becomes crucial to ensure that fish resources are protected from the adverse effects of diseases. Our results suggest the need for developing diagnostic tools to accurately identify the pathogens responsible for streptococcosis outbreaks. Monitoring hatchery and wild fish for the presence and severity of the disease will provide a better understanding for mitigating the impacts of streptococcosis.

## Methods

### Isolation and identification of bacteria

**Figure 4:**
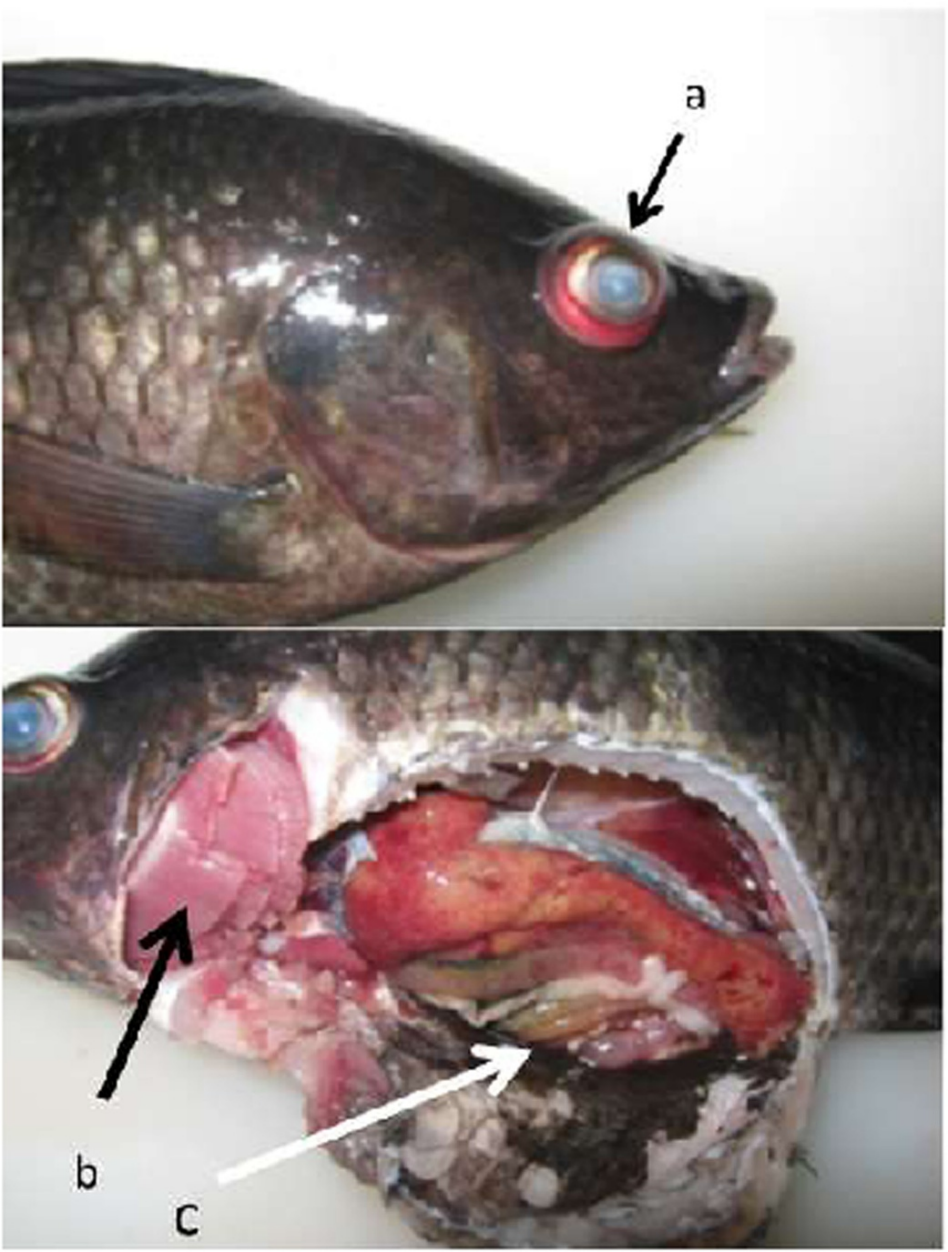
**Nile tilapia (** ***Oreochromis niloticus*** **) from Lake Sentani showing gross clinical signs of streptococcosis. a)** exophthalmus, opaque, and haemorrhagic eye, **b)** pale gill, and **c)** ascites in abdominal cavity.

**Figure 5:**
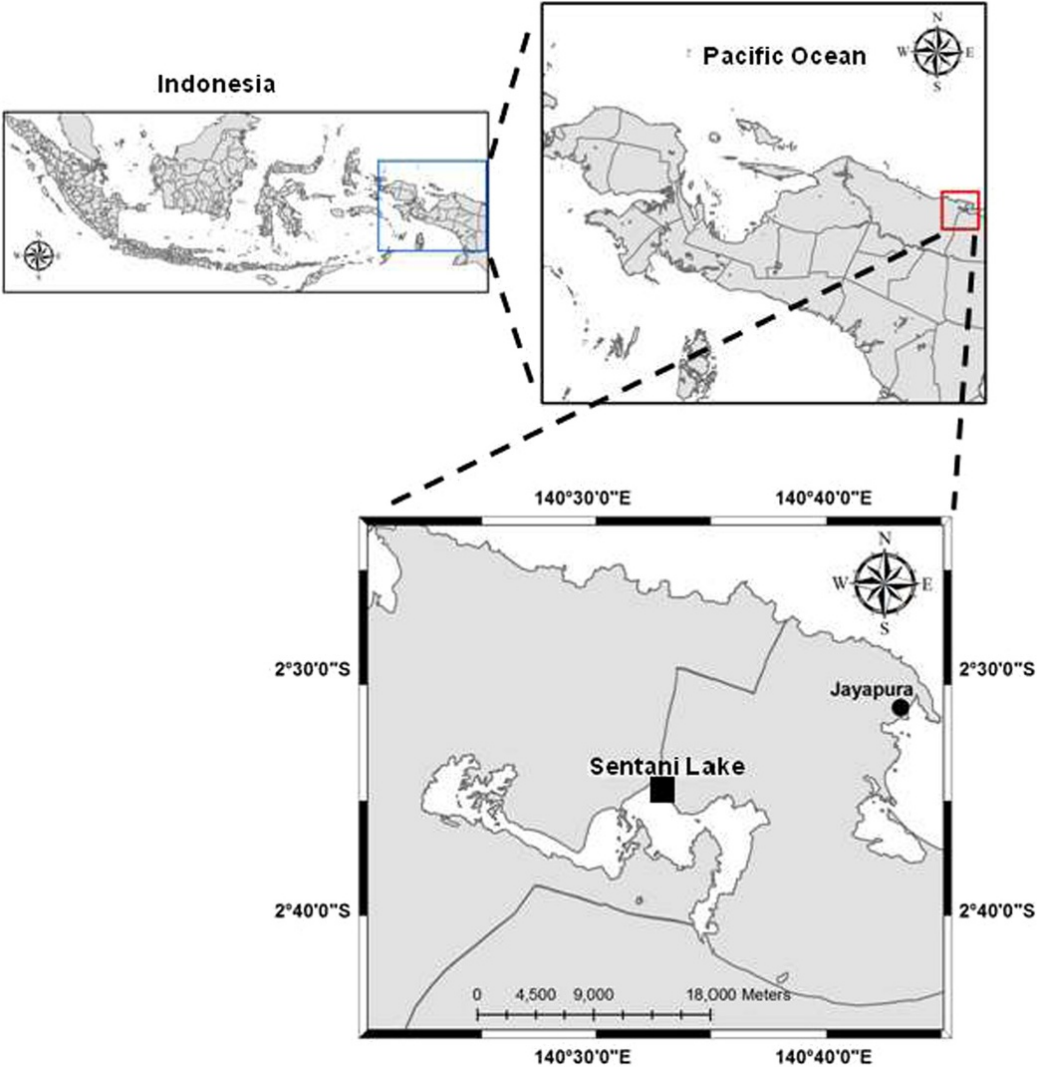
**Map of Papua showing location of floating net cage in eastern Lake Sentani, Jayapura, Papua, Indonesia.** Square mark indicates the sampling site.

Tilapia (n = 25) with a total length of 15 to 25 cm and weight of 100 to 156 g with clinical signs including pop-eye/exophthalmia, pale gills, haemorrhage in eye, erratic and circular swimming were collected from floating net cages in Lake Sentani (Figure 4). The fish were put in three plastic bags containing water from the location and transported to the Laboratory of Molecular Biology, Fish Quarantine Inspection Agency (FQIA) Sentani, Jayapura. The floating net cage is a unit from which 25 nets were suspended. Each net (3 × 3 × 3 m) was stocked with 1,000 juvenile tilapia (15–25 g), and deployed in the eastern part of Lake Sentani, Papua, Indonesia (Figure 5). The farmers purchased the tilapia seeds from a local hatchery and cultured in the cages from 3–4 months to reach a weight of about 170 – 250 g. Moribund fish are frequently observed within 1 to 2 months of culture. The average water temperature in the morning was 28.5°C at the time of sampling. In the current survey, tilapia showing clinical signs were collected at 1 to 4 months post stocking.

The fish were immediately examined in the laboratory for the presence of streptococcosis-causing bacteria. Bacteria were isolated aseptically from the kidney, brain, and eyes by streaking a loop onto Blood Agar and incubated at 35°C for 3 to 4 days. Most colonies that grew on the Blood Agar at 3 to 4 days post inoculation were small and whitish. Suspect bacterial colonies were sub-cultured onto Trypticase Soy Agar (TSA) and then subjected to morphological and biochemical tests. Colonies showing variation in biochemical tests, but were presumptively identified as *Streptococcus* according to Austin and Austin ( 2012), were subjected to PCR-sequencing in the 16S rRNA region.

### Pathogenicity test

Pathogenicity test of the representative isolates (SK, K10, P20 and M12) was conducted using healthy tilapia (average weight = 100 ± 2 g). Groups of fish (n = 6/tank) were acclimatized in aquaria (120 × 30 × 45 cm) supplied with 120 l freshwater and maintained at 35°C with aeration for about 2 weeks. The fish were fed with a commercial diet (PT Central Protein Prima) twice daily at a rate of 2% body weight. Water was 50% replaced and uneaten feed was siphoned daily. The bacteria were grown in Trypticase Soy Broth (TSB) overnight and the density of the bacterial suspension was enumerated using spread plate method on TSA. Each fish from the four groups was intraperitoneally injected with 0.1 ml of the SK, K10, P20 and M12 bacterial suspension with a mean density of 1.0 × 10^7^ CFU/mL. A control group was included where fish were injected with the same volume of sterile phosphate buffered saline (PBS). Clinical signs and morbidity were recorded daily for one week and the experiment was terminated when 100% morbidity or mortality occurred among the challenged groups. Newly dead or moribund fish were examined and tissues as described above were inoculated onto TSA. Colonies that grew on the plates were identified using biochemical tests to confirm the cause of morbidity or mortality. Kaplan-Meier survival analysis (Kaplan and Meier 1958) using SigmaPlot statistical analysis and graphing software program version 11.0 was conducted to determine the mean survival time of fish injected with suspensions of the bacterial isolates *S. iniae, S. agalactiae* and *L. garvieae* (SK, K10, P20 and M12).

### DNA extraction, PCR amplification and sequencing for 16S rRNA

The identity of the isolates was confirmed by 16S rDNA sequencing. Pure colonies of SK, K10, P20 and M12 isolates were grown overnight in TSB medium at a concentration of 10^9^ CFU/ml. One milliliter of each bacterial culture was transferred into a microcentrifuge tube and centrifuged at 7500 rpm for 10 min. The pellet was re-suspended in 100 μL buffer ATL and subjected to genomic DNA extraction following the protocols of the commercial kit (Qiagen, Gemany). The extracted gDNA was used to amplify the 16S rRNA genes using the universal primer set for prokaryotes: Forward 24f 5′-AGAGTTTGATCCTGGCT-3′, Reverse 1540r 5′-AAGGGAGGTGAT-CCAGCCGCA-3′) (Friedrich 2002). The PCR assay (20 μl) contained a final concentration of 10x buffer PCR, 0.5 mM of each primer, 2.5 U/μl of HotStar Taq DNA polymerase, 2 μl of DNA sample and nuclease free water was added to achieve the total volume of PCR mixture. The negative control utilized nuclease free water. Amplifications were carried out in a thermal cycler with an initial denaturation of 95°C for 5 min, followed by 30 cycles of 92°C for 30 sec, 52°C for 90 sec, 72°C for 1 min, and an additional final extension of 72°C for 5 min. The expected PCR product of ca. 1,500 bp was detected by electrophoresis in 1.5% agarose stained with ethidium bromide and photographed under UV light.

The PCR products from isolate SK, K10, P20 and M12 were purified using the QIAquick PCR purification kit (Qiagen, Gemany) following the manufacturer’s instructions and directly used in sequencing reaction. The purified PCR were sent to a commercial company (PT. Genetika Science Indonesia) for sequencing. Primers used for sequencing were the universal primers (24f and 1540r) as mentioned above. Additional internal primers including LF2 (AGGCAGCAGTAGGGAATC TT-3′), LF3 (5′-CTCTCTGGCCTGTAACTGAC-3′), SF2 (5′-GTGAGTGAAGAAGGT TTTCG-3′), SF3 (5′-CCATGTGTAGCGGTGAAATG-3′), and LacGarv (5′-TGGCCG ATCACCCTCTCAG-3′) were designed using Primer3 software program and used in the sequencing to generate ca. 1,500 bp nucleotide sequences. The PCR products from K10, P20, and M12 isolates were sequenced in both direction using the primers 24f and 1540r, and the internal primers LF2, LF3, and LacGarv. The SK isolates was sequenced using the primers 24f and 1540r and the internal primers SF2, SF3, and LacGarv.

### Phylogenetic analysis

The 16S rRNA gene sequences obtained from the four isolates were aligned with *Streptococcus* spp. and *Lactococcus* spp. from GenBank database using Clustal X software version 2.1. A phylogenetic analysis was performed using MEGA 6 software (Tamura et al. 2013). Neighbor-joining tree was constructed for both *Streptococcus* spp. and *Lactococcus* spp. using *Bacillus subtilis* as an out group. Gene sequences for SK, K10, P20 and M12 were deposited in GenBank under accession numbers KM209199, KM209200, KM209201 and KM209202, respectively.
